# The use of patient-specific equipoise to support shared decision-making for clinical care and enrollment into clinical trials

**DOI:** 10.1017/cts.2019.380

**Published:** 2019-06-20

**Authors:** Harry P. Selker, Denise H. Daudelin, Robin Ruthazer, Manlik Kwong, Rebecca C. Lorenzana, Daniel J. Hannon, John B. Wong, David M. Kent, Norma Terrin, Alejandro D. Moreno-Koehler, Timothy E. McAlindon

**Affiliations:** 1 Tufts Clinical and Translational Science Institute, Tufts University, Boston, Massachusetts, USA; 2 Institute for Clinical Research and Health Policy Studies, Tufts Medical Center, Boston, Massachusetts, USA; 3 School of Engineering, Tufts University, Medford, Massachusetts, USA; 4 Division of Clinical Decision Making, Tufts Medical Center, Boston, Massachusetts, USA; 5 Predictive Analytics and Comparative Effectiveness (PACE) Center, Institute for Clinical Research and Health Policy Studies (ICRHPS), Tufts Medical Center, Boston, Massachusetts, USA; 6 Division of Rheumatology, Tufts Medical Center, Boston, Massachusetts, USA

**Keywords:** Stakeholder engagement, shared decision-making, decision support, mathematical equipoise, clinical equipoise, predictive models

## Abstract

**Background:**

To enhance enrollment into randomized clinical trials (RCTs), we proposed electronic health record-based clinical decision support for patient–clinician shared decision-making about care and RCT enrollment, based on “mathematical equipoise.”

**Objectives:**

As an example, we created the Knee Osteoarthritis Mathematical Equipoise Tool (KOMET) to determine the presence of patient-specific equipoise between treatments for the choice between total knee replacement (TKR) and nonsurgical treatment of advanced knee osteoarthritis.

**Methods:**

With input from patients and clinicians about important pain and physical function treatment outcomes, we created a database from non-RCT sources of knee osteoarthritis outcomes. We then developed multivariable linear regression models that predict 1-year individual-patient knee pain and physical function outcomes for TKR and for nonsurgical treatment. These predictions allowed detecting mathematical equipoise between these two options for patients eligible for TKR. Decision support software was developed to graphically illustrate, for a given patient, the degree of overlap of pain and functional outcomes between the treatments and was pilot tested for usability, responsiveness, and as support for shared decision-making.

**Results:**

The KOMET predictive regression model for knee pain had four patient-specific variables, and an *r*^2^ value of 0.32, and the model for physical functioning included six patient-specific variables, and an *r*^2^ of 0.34. These models were incorporated into prototype KOMET decision support software and pilot tested in clinics, and were generally well received.

**Conclusions:**

Use of predictive models and mathematical equipoise may help discern patient-specific equipoise to support shared decision-making for selecting between alternative treatments and considering enrollment into an RCT.

## Introduction

The ethical and scientific basis for randomly assigning treatments in a randomized clinical trial (RCT) is the presence of clinical equipoise, the absence of a clearly superior treatment. However, this is typically not an individual patient-centered determination, but rather based on inference from groups defined by pivotal studies’ inclusion and exclusion criteria.

An alternative would be to compare patient-specific predictions of treatment outcomes, if available. If such predictions are generated by mathematical models that account for individual patient characteristics, then the potential outcomes can be compared, looking for “mathematical equipoise” [1]. Thereby, individuals could be enrolled in an RCT only when there is equipoise between treatment options based on their specific characteristics and preferences. And, if in making this determination, the treatment outcomes’ predictions are importantly different, i.e., mathematical equipoise is not present, then the patient can be offered the treatment most likely to benefit this individual. The objective is to adhere to the RCT principle of equipoise, based on patient-individualized information.

If there is the capacity to identify patient-specific equipoise embedded in electronic health records (EHRs), it could serve as a practical way in routine clinical care to detect potentially eligible patients for RCT enrollment. It also could identify those *not* appropriate for random treatment assignment, for whom this decision support could enhance care by indicating the potentially superior treatment for a given patient. This could help make transparent the basis for selection for an RCT in real time and enhance fully informed consent in the midst of clinical care. It could facilitate patient–clinician shared decision-making, both in care and in the decision to participate in an RCT.

We previously created and tested predictive instrument decision aids based on multivariable logistic regression models that provide 0%–% predictions of medical diagnoses and treatment outcomes [2–5]. These predictive instruments support decisions about hospitalization and/or treatments for acute myocardial infarction, emergency decisions substantially dominated by physician judgment, rather than complex treatment decisions that require longer patient–clinician collaboration.

Decision support for more complex and longer term decisions benefits patients when they improve knowledge, accuracy of risk perceptions, and concordance with patient values [6]. They also reduce decisional conflict due to feeling uninformed and unclear about treatment choices [6].

Shared patient–clinician decision-making is central to choosing between medical treatments and surgical total knee replacement (TKR) for long-standing knee osteoarthritis. The shared decision process involves the clinician and patient, and others as desired, sharing information about treatment options and their risks and discussing patient preferences and values [7]. Patient preferences have great relevance, as do the availability of treatments, their inconvenience and expense, and the patient’s future prospects of developing comorbidities [8]. Hampering these decisions are gaps in patient-specific evidence about alternative treatments [9] and lack of reliable measurements of clinical changes in knee osteoarthritis to allow comparisons of treatments [10]. The cross-sectional national DECISIONS survey found that more than half of patients discussing knee or hip surgery underestimated the harm from surgery, and only 28% correctly estimated the amount of pain relief following surgery [11].

Symptomatic knee osteoarthritis, estimated as affecting 17%–34% US adults [12], is the most frequent cause of dependency in lower limb tasks, especially in the elderly [13]. It contributes to 68 million work-loss days per year and over 5% of the annual retirement rate [14–17]. For many patients, as medical and physical therapy become less satisfactory, TKR is done, making it the most frequently surgically replaced joint [15], now done for 680,886 patients per year in the United States, with aggregate charges over $36 billion [18].

In this context, we sought to create the Knee Osteoarthritis Mathematical Equipoise Tool (KOMET) to be embedded in EHRs as decision support for shared clinical decision-making. KOMET was intended to identify patients for whom, based on their specific characteristics, there is no sufficient evidence to favor medical or surgical treatment. This is a circumstance in which shared patient–clinician decision-making is important – when patients’ personal preferences and objectives may dominate what might appear as a “toss-up” decision [1]. This also is the circumstance in which KOMET is intended to support shared decision-making about participation in RCTs, based on patient-specific equipoise, for practical, ethical, and targeted enrollment into RCTs.

To create the mathematical models for our prior predictive instruments, we used data from RCTs. Using data from treatments that were randomly assigned avoided the treatment effects being biased by the selection of their use among patients. Thereby, the multivariable regression models more accurately reflect the effects of treatments when used in comparable patients. However, there are many conditions and treatments for which RCT data are not available. Indeed, for the very circumstances that would call for an RCT, for which predictive models and mathematical equipoise might be wanted to assist participant selection, there often will be little RCT data on which such models could be based. Therefore, to create predictive models for this purpose, we must use data from other sources, including observational studies, registries, EHR-based data warehouses, and patient-acquired data. If these non-RCT sources could be used for creating predictive models, there would be vast opportunities for the mathematical equipoise approach to facilitate the conduct of clinical effectiveness RCTs. However, there are protean challenges and limitations.

This project sought to create KOMET as an example of the use of mathematical equipoise for determining patient-specific equipoise. As an example of this approach, we used patient-level data from existing non-RCT sources to build predictive models of treatment outcomes to determine the presence or absence of patient-specific equipoise, to inform decision-making. Demonstrating the ability to do this could support wider applicability of the mathematical equipoise method.

## Methods

To develop the KOMET’s predictive models, we created a consolidated database with treatment outcomes of knee osteoarthritis from clinical studies and patient registries. Model variables and predicted outcomes were selected based on input from stakeholders, availability of data, and variables’ contributions to models’ predictive performance. When completed, the models were incorporated into prototype decision support software and tested with clinicians and patients. Throughout, we involved stakeholders with an interest in the outcomes of the project including patients and their families, advocacy group representatives, clinicians, and researchers [19].

### Datasets

To create the modeling database, we used the following databases, which are described in more detail elsewhere [20]:

- *The Multicenter Osteoarthritis Study (MOST)*, an NIH-sponsored longitudinal, prospective, observational study of knee osteoarthritis in adults with osteoarthritis or at increased risk of developing osteoarthritis [21]. The database includes a community-based sample of 3,026 participants aged 50–79 years, with preexisting osteoarthritis or those at high risk for osteoarthritis based on weight, knee symptoms, or a history of knee injuries or operations. Approximately 60% are women, and 15% are African–Americans. Data used in this article were obtained from http://most.ucsf.edu [21].
- *The Osteoarthritis Initiative (OAI)*, an NIH-sponsored multicenter, longitudinal, prospective observational study of osteoarthritis intended as a public domain research resource that includes 4,796 men and women aged 45–79 who have, or are at high risk for developing, symptomatic knee osteoarthritis. Data were obtained from http://oai.ucsf.edu [22].
- *The New England Baptist Hospital (NEBH) Orthopedic Surgery Registry* of 2,462 patients who underwent TKR since 2011 [23]. Assessments occur prior to surgery and at 6 weeks and at 12 months and include demographic, vital signs, clinical measures, medications, knee exam, the Knee Society Score (KSS) pain and physical function score, the 12-Item Short Form Health Survey (SF-12) health status score, surgical complications, and procedure outcomes. The mean age of patients is 68 years; 57% are women.
- *The Tufts Medical Center (TMC) Orthopedic Surgery Registry* of 535 patients who had TKR surgery since 2007 [24]. Assessments occur prior to surgery, at 6 weeks, 12 months, and 24 months, including demographic, vital signs, clinical measures, medications, knee exam, pain and physical function (KSS), health status (SF-12), surgical complications, and procedure outcomes. The mean age of patients is 62 years; 61% are women.

### Predicted Clinical Outcomes

The models created on these databases predicted clinical outcomes expressed as scales; for pain, the WOMAC Index [25] and for functional status, the SF-12 Health Index, both outlined as follows [26, 27]:

- *The Western Ontario and McMaster Universities Arthritis Index (WOMAC)* [25], developed in 1982, widely used in the evaluation of hip and knee osteoarthritis. It is a self-administered questionnaire of 24 items, divided into 3 subscales: (1) pain (5 items) during walking, using stairs, in bed, sitting or lying, and standing upright; (2) stiffness (2 items) after first waking and later in the day; and (3) physical function (17 items) using stairs, rising from sitting, standing, bending, walking, getting in and out of a car, shopping, putting-on and taking-off socks, rising from bed, lying in bed, getting in and out of a bath, sitting, getting on and off the toilet, heavy domestic duties, and light domestic duties. The knee pain scale was used as the primary outcome in this project. In its raw form, the WOMAC knee pain scale ranges from 0 to 20. To make it easier to interpret and represent in the final models, we rescaled it to 0–100, with 0 representing absence of pain and 100 representing extreme pain.
- *The SF-12® Health Survey* a multipurpose “short-form” generic measure of health status [26, 27]. It was developed to be a much shorter, yet valid, alternative to the SF-36® for use in large surveys of general and specific populations and for large longitudinal studies of health outcomes. We used its physical functioning summary score as the second predicted outcome for this project. The SF-12 scores range from 0 to 100, with higher scores indicating better function [28].

### Creating the Modeling Database

The database for creating KOMET models included two types of registries. MOST and OAI had data collected on knee osteoarthritis at fixed intervals per their protocols, including those who underwent TKR and were continued to be followed. The other two registries, NEBH and TMC, were from hospitals that collected baseline and follow-up data only on their patients who had TKR.

For KOMET, our target patients were those with knee osteoarthritis who had reached the clinical state at which they would be making a decision of whether or not to have TKR. Lacking a cohort of such patients randomized to medical or surgical options, we used data from patients who had TKR, and matched them to patients (knees) who did not have TKR, but otherwise had similar characteristics. To do this, we created a database in which the “knee” was used as the unit of analysis, and matching was done based on characteristics of the knee and the patient. Thereby, we created a study sample of pairs of similar patients who could be considering this therapeutic choice.

For the MOST and OAI registries, we identified all knees that underwent TKR and then used data collected at the *closest previous* visit as the “baseline” visit for that TKR including demographics, knee characteristics, comorbidities, mental and physical function, and other clinical features. To find non-TKR “control knees,” we created a subdatabase of all knee visits of all patients, excluding any that occurred after a TKR. We then used a “greedy” matching computer algorithm [29] to select control knees for each “TKR knee” (within the same database). Thereby, each TKR knee in OAI was matched to a similar non-TKR knee from OAI, based on matching variables at baseline. Because the TMC and NEBH samples only included TKR subjects, we drew their matched non-TKR controls from a pooled dataset of knee visits from the OAI and MOST registries.

The variables used for matching differed among the databases, based on data availability. As a guide to determine variables to use for matching, we used input from the research team, clinicians, stakeholders, and the literature [30]. For matching, we converted continuous variables to categories. We did not always require exact matches because we did not want to lose patients who had TKR from the model-building sample, and we could statistically adust for differences between the TKR and non-TKR groups in the modeling process.

### Creating Predictive Models for Outcomes

Analyses were done using SAS for Windows, version 9.4 TS Level 1M2, Copyright © 2002-2012 by SAS Institute, Cary, NC and SAS Enterprise Guide, version 7.13 HF3 (Copyright SAS Institute, Cary NC, USA). More details of modeling can be found in a separate article [20].

We developed a multivariable linear regression model for the 1-year knee pain outcome based on the WOMAC score, or when a database lacked WOMAC items, using an estimated WOMAC score. Our approach was to develop the model using a set of matched TKR to non-TKR knees from the OAI database, then to validate/test it on a set of matched TKR and non-TKR knees from the MOST database, and then update it by pooling the OAI and MOST datasets and building a new model, starting with variables used in the model developed in the OAI data and tested on the MOST data. We also rederived models, using a similar variable selection process, but with a more limited set of candidate predictor variables, in order to include NEBH and Tufts datasets together with OAI and MOST. This entire process was repeated for the 1-year functional outcome (SF-12 physical component score). To create models that could provide predicted estimates of 1-year knee pain and 1-year function, with and without TKR, for any patient based on their characteristics, all models included a treatment indicator (interaction) variable. Covariates and interactions of treatment with covariates were explored in the different phases of the modeling process. No adjustment for matching was done in the linear regression during modeling. This was because the purpose of matching was to create a reasonably balanced study sample of patients who either had TKR or did not have TKR with comparable characteristics, with covariates in the models accounting for remaining imbalances between the groups [31].

### Prototype Decision Support Software Development and Usability Testing

The goal of software development and usability testing was to translate the results of the predictive models into easily understood, patient-specific reports that could be produced in the course of clinical care for shared treatment decision-making and, if appropriate, enrollment into an RCT.

*Decision Support Software Development*. There were two KOMET software development tasks: one for the analytics and another for the user interface. *Analytics* development included implementing the predictive models as reusable, multiplatform software components to generate the current and 1-year-predicted pain and function outcomes for nonsurgical and surgical treatments. The analytics software also calculated the respective 95% confidence intervals around each prediction as the basis for considering the degree of overlap that would suggest near equivalence or patient-specific equipoise. *User interface* development included creating a web browser-based questionnaire interface to collect patient demographics, items for computing the WOMAC pain score, the SF-12 physical functioning scale, and comorbidities. Together, the analytics and user interface components included methods for data retention and presentation of the predicted outcome results.

Then, the predictive models then were incorporated into the web-based decision support application for iterative user testing. Usability testing included a “think-aloud” protocol and a usability-testing script administered by research staff. The IRB determined that the project was exempt from IRB review.

### Depiction of Pain and Function Predictions for Nonsurgical Treatment versus TKR and Patient-Specific Equipoise

**Fig. 1.**
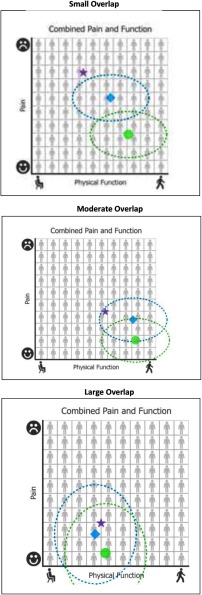
Three sample graphs with small, moderate, and large amounts of “uncertainty circle” overlap.

Mathematical equipoise was defined for KOMET as when the pain and functioning outcome predictions with nonsurgical care and TKR are close, i.e., within, or overlapping, each other’s “uncertainty circle.” In Fig. 1, this is illustrated by hypothetical example graphs with small, moderate, and large amounts of “uncertainty circle” overlap. The uncertainty circle is defined by the shaded area extending around each of the point estimates and is derived from the 95% prediction intervals associated with the predictions for the two outcomes. The blue diamond represents the outcome prediction point estimate for nonsurgical care; the green circle represents the point estimate for TKR. The points are plotted on an X–Y plane with function on the x-axis and pain on the y-axis. The large shaded blue and green overlapping circles around the 95% prediction intervals of the pain and function point estimates and represent the uncertainty associated with the individual predictions. We computed the mathematical distance between the nonsurgical and TKR predictions as the distance between the two coordinates on the pain and function graph using the following equation:

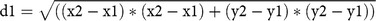

where the coordinate for pain and function predictions with nonsurgical care is represented as (x1, y1) and the coordinate for pain and function predictions with TKR as (x2, y2).

## Results

### Study Design and Database Creation

The final database included 1,452 knees (726 with TKR and 726 without) of 1,322 patients. Ninety-one percent of patients (1,204) had a single knee included in the database, 8% (106) had two knees used or a single knee used two times, and 1% (12) had their knees used three times. OAI TKR knees were matched to control knees from OAI, and MOST TKR knees were matched to controls from MOST. Because NEBH and TMC included only TKR knees, their controls were drawn from non-TKR knees from OAI and MOST. In the final matched database, the relative contributions of TKR knees were OAI, 252; MOST, 154; NEBH, 248; and TMC, 72. For the control knees, contributions were OAI, 472, and MOST, 254.

### Study Sample

Characteristics of the study sample are summarized in Table 1. Approximately 60% were women, the mean age was 65 years, and the mean body mass index (BMI) was 31 kg/m^2^. On the 0–100 pain scale (100 indicating extreme pain), the mean baseline knee pain was significantly higher in the TKR than non-TKR group (mean = 45.6 vs. 40.5, *P* < 0.01), despite efforts to match on this variable (categorized). Baseline characteristics of interest to clinicians and stakeholders that were considered for the modeling process were comparable between TKR and non-TKR knees, and also for the variables used in the final models, using imputed data. Irrespective of significance, all variables listed in Table 1 were used in building the multivariable models of the clinical outcome scales.

**Table 1.** Description of pooled study sample used for model derivation for N = 1,452 matched knees (imputed data); TKR = Total Knee Replacement; WOMAC = The Western Ontario and McMaster Universities Arthritis Index

Variable	TKR (n = 726) Mean ± SD	Non-TKR (n = 726) Mean ± SD	TKR minus non-TKR Delta (Δ) and [95% CI]	Effect size*(Δ/SD)
Baseline characteristics				
Age	65.29 ± 8.57	64.77 ± 8.57	0.52 [−0.36, 1.40]	0.03
Male, *N* (%)	0.43 ± 0.49	0.42 ± 0.49	0.00 [−0.05, 0.06]	0
Baseline BMI	31.31 ± 6.49	30.97 ± 6.36	0.34 [−0.32, 1.00]	0.03
Baseline SF-12: Physical	37.16 ± 9.46	38.59 ± 10.94	−1.44 [−2.49, −0.38]	−0.07
Baseline SF-12: Mental	52.56 ± 11.48	53.62 ± 11.82	−1.07 [−2.27, 0.13]	−0.05
Baseline WOMAC knee pain (0–100)	45.59 ± 21.87	40.48 ± 21.76	5.11 [2.87, 7.36]	0.12
Baseline knee pain, contralateral (0–100)	18.92 ± 21.06	19.66 ± 22.05	−0.74 [−2.96 , 1.48]	−0.02
Baseline hip pain or pain/ache/stiff	0.34 ± 0.50	0.62 ± 0.51	−0.27 [−0.33 , −0.22]	−0.27
At least one comorbidity, N (%)	0.32 ± 0.52	0.31 ± 0.56	0.01 [−0.05, 0.07]	0.01
Narcotics, N (%)	0.14 ± 0.36	0.13 ± 0.36	0.00 [−0.03, 0.04]	0.01
Follow-up results				
Follow-up SF-12: Physical	44.48 ± 11.88	39.81 ± 10.80	4.67 [3.51, 5.84]	0.21
Follow-up WOMAC knee pain (0–100)	13.92 ± 19.44	29.22 ± 19.45	−15.30 [−17.30, −13.30]	−0.39

* Shaded rows indicate variables where definitions varied between databases so that these variables ultimately were excluded as candidates in the building of final models.

TKR = Total Knee Replacement; WOMAC = The Western Ontario and McMaster Universities Arthritis Index

Comparisons of mean SF-12 scores between TKR and non-TKR groups showed better physical and mental function in the non-TKR groups than TKR groups, with the difference being significant for physical function (mean = 37.2 vs. 38.6, *P* = 0.008). Overall, at follow-up, there was less knee pain and better physical function in the TKR than the non-TKR groups. Distributions of variables used for the matching process confirmed that in each database, characteristics used for matching were well balanced between the TKR and non-TKR knees.

### Model Development

Linear regression was used to model the two outcomes the WOMAC knee pain scale (rescaled 0– 100) and the SF-12 physical functioning component score. Based on extensive discussions with patient and clinician stakeholders, 1 year was chosen as the target follow-up time to have a time point beyond recovery from surgery (estimated as up to 9 months). Stakeholders felt benefits of surgery were stable beyond that time point, and so to address data inconsistencies and gaps, data from up to 5 years past baseline were allowed for use when no closer follow-ups were available.

### Summary of Multivariable Models

The final predictive models for pain and physical functioning are presented in Table 2.

**Fig. 2.**
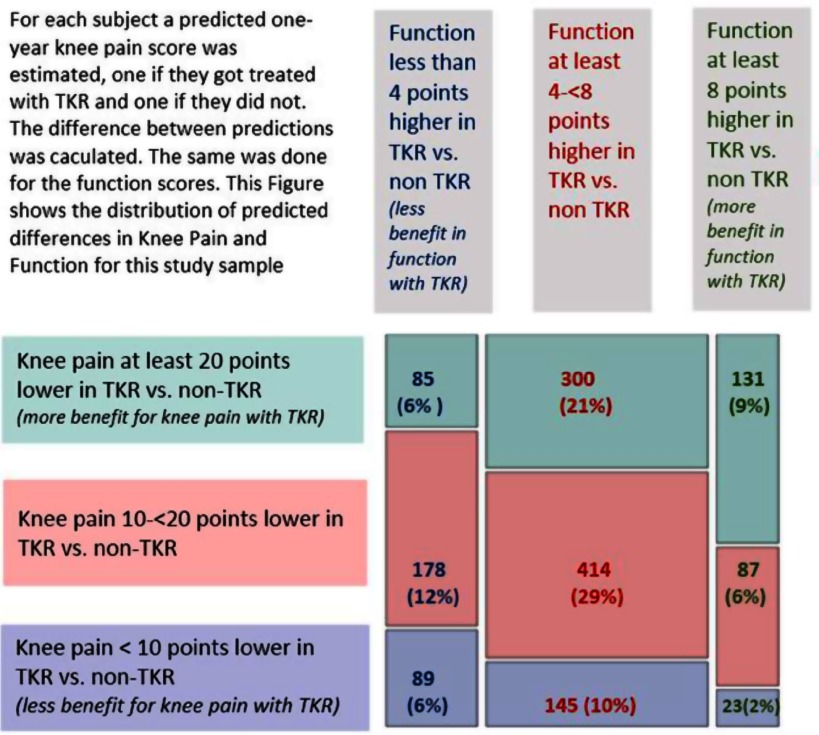
Mosaic plot showing distribution of predicted differences (TKR vs. non-TKR) for 1-year knee pain and SF-12 physical function in pooled data (N = 1,452 subjects).

We used these models to predict 1-year knee pain and physical functioning for the treatment each subject underwent (TKR or non-TKR), and also for the counterfactual (alternative) treatment. These data allowed us to predict the difference in pain and function outcomes for each patient for TKR and non-TKR. The distribution of predicted differences is in Fig. 2. The figure shows that there was a range of predicted improvement with TKR, and those patients predicted to have benefit in knee pain may not be the same as those predicted to have benefit in physical functioning. Nine percent of subjects had a predicted gain in function of at least 8 SF-12 physical function points and a predicted reduction in knee pain of at least 20 points (on WOMAC scale of 0–100). At the other end of the spectrum, 6% had predicted gains in physical function of less than 4 points and reduction of knee pain of less than 10 points. Only 2% had larger gains in physical function and smaller improvements in pain. For illustrative purposes, Fig. 2 also shows sample subjects from each of nine combinations of estimated knee pain and physical function change. Examples of subjects with the most, intermediate, and least estimated reduction of pain and gain in function, with 95% prediction intervals for the estimates, are shown in Table 3. Subjects with higher baseline knee pain had the largest predicted reductions in knee pain with TKR compared to non-TKR. Younger patients with lower SF-12 scores had the largest predicted benefits in physical function with TKR versus not having TKR. These differences in estimated benefit between subjects are a result of the interaction terms included in the multivariable models.

**Table 2.** Final models for 1-year knee pain (P2) and SF-12 physical function (F2)

Term in model, status at baseline	Range in dataset (5th–95th percentile)	P2: knee pain model (higher scores mean more knee pain)	F2: physical function (SF-12) (higher scores mean better function)
		Adjusted *r*^2^ = 0.32	Adjusted *r*^2^ = 0.34
		Beta coeff (std err), *P* value*	Beta coeff (std err), *P* value
Model intercept (constant)		31.44 (5.52), *P* < .0001	17.40 (4.27), *P* < .0001
Treatment (1 = TKR, 0 = control)		−3.33 (2.16), *P* = 0.1246	25.41 (4.33), *P* < .0001
WOMAC knee pain (base), 100 point scale	10–80	0.49 (0.03), *P* <.0001	
Interaction: treatment × WOMAC knee pain		−0.33 (0.05), *P* <.0001	
Age, years	51–79	−0.12 (0.05), *P* = 0.0225	−0.05 (0.04), *P* = 0.2397
SF-12 mental component [base]	34–66	−0.11 (0.05), *P* = 0.033	0.19 (0.04), *P* <.0001
SF-12 physical component [base]	23–53	−0.21 (0.07), *P* = 0.0017	0.55 (0.03), *P* <.0001
Gender (1 = male, 0 = female)	42% male		0.99 (0.57), *P* = 0.0873
Body mass index, kg/m^2^	23–41		−0.19 (0.05), *P* = 0.0008
Charlson comorbidity score ≥ 1 (vs. 0)	31% with at least 1		−2.05 (0.60), *P* = 0.0009
Interaction: treatment × age			−0.15 (0.06), *P* = 0.0084
Interaction: treatment × SF-12 mental score			−0.18 (0.06), *P* = 0.0013

Beta coefficients, standard errors, and *P* values are from combined linear regression models built on an imputed dataset

**Table 3.** Estimated outcomes for a sample of cases

Predicted change with TKR compared to non-TKR		Baseline characteristics							Knee pain (1 year): estimate and 95% prediction interval			SF-12 function (1 year): estimate and 95% prediction interval		
Estimated reduction in knee pain	Estimated improvement in function	Gender	Age	BMI	Any comorbidities	SF-12 mental	WOMAC knee pain	SF-12 physical	Non-TKR	TKR	TKR minus non- TKR	Non-TKR	TKR	TKR minus non-TKR
Most pain reduc (≥20 pt)	Most gain fcn (≥8 pt improve)	F	58	33.3	N	38	65	30	46 (12 to 80)	21 (80 to −13)	−24.5 (−72.4 to 23.5)	32 (15 to 49)	42 (49 to 25)	9.6 (−14.6 to 33.8)
Most pain reduc (≥20 pt)	Mid gain fcn (4−<8 pt improve)	F	63	35.0	N	60	70	41	43 (09 to 77)	17 (77 to −17)	−26.1 (−74.1 to 21.9)	42 (25 to 59)	47 (59 to 30)	4.7 (−19.5 to 28.8)
Most pain reduc (≥20 pt)	Least gain fcn (<4 pts improve)	M	77	24.3	Y	60	55	36	35 (01 to 69)	14 (69 to −20)	−21.2 (−69.2 to 26.7)	39 (22 to 56)	42 (56 to 25)	2.7 (−21.5 to 26.9)
Mid pain reduc (≥10 to <20 pts)	Most gain fcn (≥8 pt improve)	M	63	28.3	N	34	45	36	34 (00 to 68)	16 (68 to −17)	−18.0 (−65.9 to 30.0)	36 (19 to 53)	46 (53 to 29)	9.6 (−14.6 to 33.8)
Mid pain reduc (≥10 to <20 pts)	Mid gain fcn (4−<8 pt improve)	M	66	31.3	Y	62	25	50	18 (−16 to 52)	7 (52 to −27)	−11.5 (−59.4 to 36.5)	46 (29 to 63)	50 (63 to 33)	4.0 (−20.2 to 28.2)
Mid pain reduc (≥10 to <20 pts)	Least gain fcn (<4 pts improve)	M	71	35.8	Y	59	30	44	22 (−12 to 55)	8 (55 to −25)	−13.1 (−61.0 to 34.8)	42 (25 to 59)	46 (59 to 29)	3.7 (−20.4 to 27.9)
Least pain reduc (<10 pts)	Most gain fcn (≥8 pt improve)	M	51	23.4	N	39	20	49	20 (−14 to 54)	11 (54 to −23)	−9.8 (−57.9 to 38.2)	46 (29 to 63)	56 (63 to 39)	10.5 (−13.7 to 34.8)
Least pain reduc (<10 pts)	Mid gain fcn (4−<8 pt improve)	F	62	26.3	N	61	20	44	18 (−16 to 52)	8 (52 to −26)	−9.8 (−57.8 to 38.1)	45 (28 to 62)	50 (62 to 33)	4.7 (−19.5 to 28.8)
Least pain reduc (<10 pts)	Least gain fcn (<4 pts improve)	F	74	27.4	Y	56	5	51	8 (−26 to 42)	3 (42 to −31)	−5.0 (−53.0 to 43.1)	46 (28 to 63)	49 (63 to 32)	3.7 (−20.4 to 27.9)

### Prototype Decision Support Software Development, Interface Design, and Usability Testing

The KOMET development process resulted in the creation of one web-based application for clinicians (http://medicalequipoise.com/tkrclinician) and one for patients (http://medicalequipoise.com/tkrpatient). Both applications are composed of an analytics software library that also could be embedded into an EHR system.

**Fig. 3.**
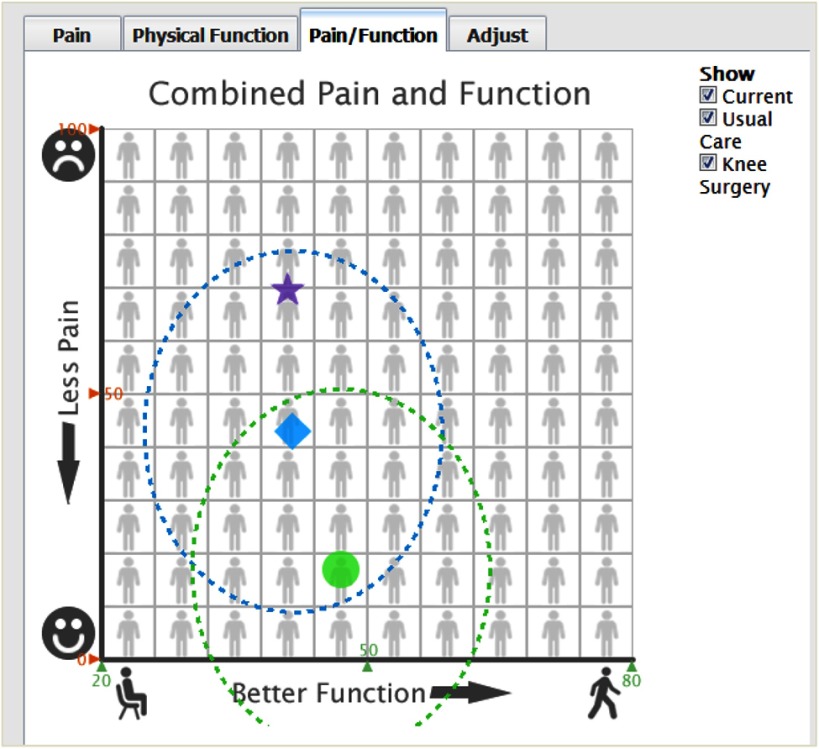
Early combined pain and function predicted outcome results page.

**Fig. 4.**
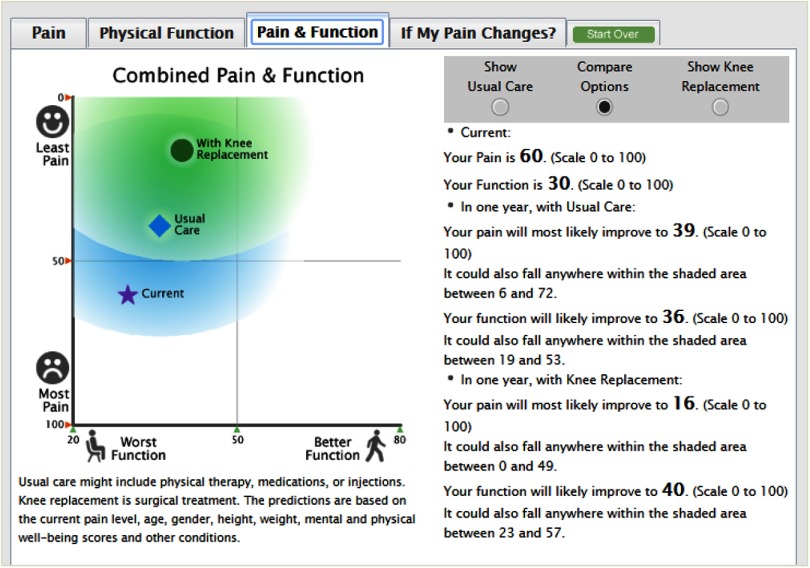
Final combined pain and function predicted outcome results page.

Based on the mathematical equipoise approach and the uncertainty estimates around the predictions, we developed for KOMET a way to identify patients for whom enrollment in a RCT might be appropriate. We defined mathematical equipoise as when predictions of pain and functioning outcomes with nonsurgical care and TKR are relatively close and their uncertainty circles around each of the point estimates depicting the 95% limits of the predictions overlap on a two-dimensional graph of pain and function (see Figs. 3 and 4). As outlined above, we computed the mathematical distance between the nonsurgical and TKR predictions as the distance between the two coordinates on the pain and function graph. Empirically, a distance of less than or equal to 20 was used to flag the presence of equipoise. When mathematical equipoise was present, an alert appeared on the software’s results page and a patient contact and screening form was generated to initiate a clinical trial recruitment conversation. When we asked patients about usefulness of the information for decision-making, each stated that the tool was helpful or somewhat helpful. All wanted to discuss the results with their physician.

## Discussion

This project created KOMET, which uses mathematical models that predict patient-specific outcomes of treatment options to detect patient-specific equipoise between nonsurgical and surgical TKR. To support decisions both for clinical care and RCT enrollment, KOMET’s graphical software is intended for use in EHRs during routine clinical care. When the patient-specific predictions of outcomes between the two treatment options are not importantly different, suggesting clinical equipoise for that patient, enrollment in an RCT that compares the treatments can be considered. Alternatively, when the predictions suggest that one treatment is likely to have a better outcome for that patient, trial enrollment would not be appropriate, but the identification of the potentially superior treatment can inform patient–clinician decision-making. Thereby, this approach for enrolling RCT participants also can support clinical decision-making for those not to be enrolled in an RCT.

For the use of patient-specific mathematical equipoise to help fill in gaps in RCTs, predictive models for the target conditions will need to be built on non-RCT data, e.g., from clinical registries, EHRs, and other sources. As an example, this project applied this approach to treatment question on which there were essentially no prior RCTs, but one for which such evidence would be important for patients and society.

To build KOMET, we created a consolidated database from non-RCT sources on which we created predictive models of the outcomes of surgical TKR and nonsurgical treatments. Informed by patients and clinicians views on the representation of pain and functional outcomes, we developed multivariable mathematical models that predict patient-specific outcomes of surgical and nonsurgical treatment. We used statistical and analytic methods to adjust, to the extent possible, for the inherent biases in the databases – and a variety of analyses were done to understand how to best model and represent the predicted outcomes. We incorporated these models into an stakeholder-informed prototype decision support software for potential incorporation into EHRs. Thereby, the KOMET is intended to exemplify a tool that is responsive to the perspectives and needs of patients and clinicians in supporting shared decision-making both for RCT enrollment and treatment.

We think this approach could be useful for comparative effectiveness research (CER). The impact of CER is based on evidence generation, which then leads to evidence synthesis, interpretation, application, dissemination, implementation in widespread practice, and then feedback for the generation of new evidence. Ideally, this entire chain rests upon having unbiased and generalizable RCT evidence. An EHR-based method for patient-centered enrollment into RCTs should allow more targeted comparative effectiveness trials in more diverse clinical sites and more representative participants, thereby applicable to more patients and more care settings. Moreover, more complete EHR-based identification of potential participants could reduce clinical trial duration, bringing results to the public sooner and reducing costs.

Aside from these potential benefits, we hope that this method might facilitate overall care by promoting conversations between clinicians and patients based on evidence specific to their care. This also could facilitate clinicians’ and the public’s understanding of, and participation in, clinical trials.

Although not ready for widespread implementation, in its content, user interface, and connectability to EHRs, KOMET did function as intended. We believe it warrants further development for implementation in clinical settings, even while KOMET has limitations related to the available data, modeling methods, analytic methods, and the prototype software.

An important limitation of our approach is that the models were created on potentially biased data. We sought data from studies that had both surgical and medical treatment of knee osteoarthritis, and two of our studies had that, but two other registries included only one treatment (surgery). Both types of sources provide challenges for creating comparable patients who underwent the two treatments, as is needed to make accurate models of the two treatments. However, for mathematical equipoise to serve its intended purpose of facilitating RCTs of treatments for which none have yet been done, its models will need to be made on non-RCT data, as was the case in this project. We undertook many checks to accurately represent effects despite the likely biased samples. KOMET’s models performed well despite this challenge, but for wider use of this approach, additional sources of data and analytic methods should be developed.

The modeling methods also have limitations. Multivariable regression methods we used have advantages over some more computer-intensive methods, including their clearly interpretable variable coefficients and resistance to overfitting, compared with some computer-driven methods [32]. However, larger databases on which more corrections might be made (e.g., by propensity scores) and newer computer methods might have advantages in terms of performance and ease of development and should be explored.

In addition to the modeling methods, the variables we used have limitations. Based on the collection of important variables in the available databases, published clinical evidence, and stakeholder input, we believe we used very credible variables to represent independent and dependent (treatment outcome) variables. However, there is a specific limitation in the functional outcome we predicted, which reflected our intent to capture a holistic physical function of the patient, based on the SF-12 functional scale. In looking at the results of the KOMET’s predictions, we noticed that pain is often very substantially changed by surgery, but function tends to have a relatively modest improvement. In discussing this with patients, it appears possible that we might have better captured their meaningful knee functional improvement if we had used a more knee-specific function rather than overall physical functioning. Meetings with stakeholders suggest that both overall and knee-related function are important, and future work should develop predictions of the more specific knee-related function would be useful to both patient and clinical stakeholders [33, 34]. There are examples in other diseases in which, for specific conditions, disease-specific outcomes are more useful than more general functional outcomes [35, 36]. Additional research that uses a more specific functional outcome seems warranted. In addition, ideally, this approach could be expanded to incorporate outcomes of harm or different weightings of efficacy and safety outcomes, which would presumably enhance its utility.

We believe the basis for patient-specific equipoise, mathematical equipoise will benefit from further development, including a generalizable method by which the overlap of two patient-specific predictions can be designated as sufficient to constitute equipoise. In addition, as we learned from our clinical testing, ways to effectively convey the meaning of patient-specific equipoise deserve development such that is clear to patients and clinicians.

The prototype KOMET software has limitations. The creation of full-featured, user-friendly, robust software was beyond the scope of this project. Our prototype needs significant further development before it could be used in routine care. Nonetheless, we believe that it is quite attractive and functional, and in the context of its intended role in this project, a successful product of this project. More research is needed for this type of decision support, which should be full-featured, user-friendly, interoperable, and robust, and will be attractive to help identify patients for whom enrollment in an RCT might be appropriate.

The limitations listed above all suggest areas for future research. Approaches must be developed that lessen the biases inherent in clinical registry data. Just having more data, such from EHR data warehouses and other sources, will not eliminate biases. Finding ways to mitigate the biases, using selection and sampling methods and other approaches, will be extremely important for work on mathematical equipoise, and for many other efforts to harvest clinically important insights from clinical data.

Modeling clinical outcomes based on data is evolving rapidly, and increasingly sophisticated computer-based methods, such as artificial intelligence and machine learning, are being applied to analysis of clinical data [37]. Although computer-based algorithms have had tendencies to over-fit [32], which can limit generalizability to new populations, these methods are advancing, and an investigation of best methods is certainly warranted.

## Conclusions

This project demonstrated the use of predictive instruments and mathematical equipoise as a method for detecting patient-specific equipoise between alternative treatments. Embedded in EHRs, this approach should help identify patients for whom one or the other treatment seems likely to yield better outcomes based on their specific characteristics, and also patients for whom there is insufficient evidence to favor one treatment. This can be part of a shared decision-making process that incorporates the patient’s preferences and priorities, and it also, as clinical equipoise, can support enrollment into an RCT. This approach will require further development, and will benefit from testing in clinical practice and for facilitating comparative effectiveness trials.

## References

[1] Selker HP, et al. Random treatment assignment using mathematical equipoise for comparative effectiveness trials. Clinical and Translational Science 2011; 4(1): 10–16. 21348950 10.1111/j.1752-8062.2010.00253.x PMC3076795

[2] Kent DM, et al. A percutaneous coronary intervention-thrombolytic predictive instrument to assist choosing between immediate thrombolytic therapy versus delayed primary percutaneous coronary intervention for acute myocardial infarction. The American Journal of Cardiology 2008; 101(6): 790–795. 18328842 10.1016/j.amjcard.2007.10.050

[3] Selker HP, et al. Use of the acute cardiac ischemia time-insensitive predictive instrument (ACI-TIPI) to assist with triage of patients with chest pain or other symptoms suggestive of acute cardiac ischemia. A multicenter, controlled clinical trial. Annals of Internal Medicine 1998; 129(11): 845–855. 9867725 10.7326/0003-4819-129-11_part_1-199812010-00002

[4] Selker HP, et al. Patient-specific predictions of outcomes in myocardial infarction for real-time emergency use: a thrombolytic predictive instrument. Annals of Internal Medicine 1997; 127(7): 538–556. 9313022 10.7326/0003-4819-127-7-199710010-00006

[5] Selker HP, Beshansky JR, Griffith JL, Investigators TPIT. Use of the electrocardiograph-based thrombolytic predictive instrument to assist thrombolytic and reperfusion therapy for acute myocardial infarction. A multicenter, randomized, controlled, clinical effectiveness trial. Annals of Internal Medicine 2002; 137(2): 87–95. 12118963 10.7326/0003-4819-137-2-200207160-00006

[6] Stacey D, et al. Decision aids for people facing health treatment or screening decisions. The Cochrane Database of Systematic Reviews 2014; (1): CD001431. 10.1002/14651858.CD001431.pub4 24470076

[7] Charles C, Gafni A, Whelan T. Shared decision-making in the medical encounter: what does it mean? (or it takes at least two to tango). Social Science & Medicine 1997; 44(5): 681–692. 9032835 10.1016/s0277-9536(96)00221-3

[8] Selten EM, et al. Reasons for treatment choices in knee and hip osteoarthritis: a qualitative study. Arthritis Care & Research 2016; 68(9): 1260–1267. 26814831 10.1002/acr.22841

[9] Weng HH, et al. Development of a decision aid to address racial disparities in utilization of knee replacement surgery. Arthritis and Rheumatism 2007; 57(4): 568–575. 17471558 10.1002/art.22670

[10] Eyles JP, et al. Can we predict those with osteoarthritis who will worsen following a chronic disease management program? Arthritis Care & Research 2016; 68(9): 1268–1277. 26749177 10.1002/acr.22836

[11] Fagerlin A, et al. Patients’ knowledge about 9 common health conditions: the DECISIONS survey. Med Decis Making 2010; 30(5 Suppl): 35S–52S. 20881153 10.1177/0272989X10378700

[12] Lawrence RC, et al. Estimates of the prevalence of arthritis and other rheumatic conditions in the United States. Part II. Arthritis and Rheumatism 2008; 58(1): 26–35. 18163497 10.1002/art.23176 PMC3266664

[13] Guccione AA, et al. The effects of specific medical conditions on the functional limitations of elders in the Framingham Study. American Journal of Public Health 1994; 84(3): 351–358. 8129049 10.2105/ajph.84.3.351 PMC1614827

[14] HJ M. Clinical features of osteoarthritis In: Kelly WN, Harris ED, Ruddy S, Sledge CB, eds. Textbook of Rheumatology. 4th Ed. Philadelphia, PA: W.B. Saunders Co.; 1993;1374–1384.

[15] *The Incidence and Prevalence Database for Procedures* Sunnyvale, CA: Timely Data Resources; 1995.

[16] Kosorok MR, et al. Restricted activity days among older adults. American Journal of Public Health 1992; 82(9): 1263–1267. 1503169 10.2105/ajph.82.9.1263 PMC1694318

[17] Kramer JS, Yelin EH, Epstein WV. Social and economic impacts of four musculoskeletal conditions. A study using national community-based data. Arthritis and Rheumatism 1983; 26(7): 901–907. 6223644 10.1002/art.1780260712

[18] HCUPnet. Healthcare Cost and Utilization Project U.S. Department of Health & Human Services 2014. Retrieved from https://hcupnet.ahrq.gov/#setup. Accessed April 25, 2017.

[19] Deverka PA, et al. Stakeholder participation in comparative effectiveness research: defining a framework for effective engagement. Journal of Comparative Effectiveness Research 2012; 1(2): 181–194. 22707880 10.2217/cer.12.7 PMC3371639

[20] Ruthazer R, et al. *Development of Predictive Models for Use with Shared Decision Support Tool: Case Study*. In progress.

[21] Multicenter Osteoarthritis Study (MOST). 2009 Retrieved from http://most.ucsf.edu/. Accessed October 5, 2018.

[22] The Osteoarthritis Initiative (OAI). 2013 Retrieved from https://data-archive.nimh.nih.gov/oai/. Accessed October 5, 2018.

[23] Richmond J. New England Baptist Hospital (NEBH) Orthopedic Surgery Registry. 2004. Boston, Massachusetts, USA.

[24] Tufts Medical Center (TMC) Orthopedic Surgery Registry. 2007. Boston, Massachusetts, USA.

[25] Bellamy N. WOMAC Osteoarthritis Index. Retrieved from http://www.womac.org/womac/index.htm. Accessed February 22, 2017.

[26] Ware JEJ. SF-12 Health Survey. Retrieved from http://www.outcomes-trust.org/instruments.htm#SF-12. Accessed February 22, 2017.

[27] Ware J, Jr., Kosinski M, Keller SD. A 12-Item Short-Form Health Survey: construction of scales and preliminary tests of reliability and validity. Medical Care 1996; 34(3): 220–233. 8628042 10.1097/00005650-199603000-00003

[28] Lacson E, Jr., et al. A comparison of SF-36 and SF-12 composite scores and subsequent hospitalization and mortality risks in long-term dialysis patients. Clinical Journal of the American Society of Nephrology 2010; 5(2): 252–260. 20019120 10.2215/CJN.07231009 PMC2827595

[29] Kosanke J, Erik B. GMATCH Macro for Greedy Matching. Retrieved from http://www.mayo.edu/research/departments-divisions/department-health-sciences-research/division-biomedical-statistics-informatics/software/locally-written-sas-macros. Accessed February, 2014.

[30] Riddle DL, Kong X, Jiranek WA. Factors associated with rapid progression to knee arthroplasty: complete analysis of three-year data from the osteoarthritis initiative. Joint Bone Spine 2012; 79(3): 298–303. 21727020 10.1016/j.jbspin.2011.05.005

[31] Stuart EA. Matching methods for causal inference: a review and a look forward. Statistical Science 2010; 25(1): 1–21. 20871802 10.1214/09-STS313 PMC2943670

[32] Selker HP, et al. A comparison of performance of mathematical predictive methods for medical diagnosis: identifying acute cardiac ischemia among emergency department patients. Journal of Investigative Medicine. 1995; 43(5): 468–476. 8528758

[33] Brazier JE, et al. Generic and condition-specific outcome measures for people with osteoarthritis of the knee. Rheumatology 1999; 38(9): 870–877. 10515649 10.1093/rheumatology/38.9.870

[34] Bombardier C, et al. Comparison of a generic and a disease-specific measure of pain and physical function after knee replacement surgery. Medical Care 1995; 33(4 Suppl): AS131–AS144. 7723441

[35] Binkley JM, et al. The Lower Extremity Functional Scale (LEFS): scale development, measurement properties, and clinical application. North American Orthopaedic Rehabilitation Research Network. Physical Therapy 1999; 79(4): 371–383. 10201543

[36] Patrick DL, Deyo RA. Generic and disease-specific measures in assessing health status and quality of life. Medical Care 1989; 27(3 Suppl): S217–S232. 2646490 10.1097/00005650-198903001-00018

[37] Jiang F, et al. Artificial intelligence in healthcare: past, present and future. Stroke and Vascular Neurology 2017; 2(4): 230–243. 29507784 10.1136/svn-2017-000101 PMC5829945

